# Latent Class Analysis of Subphenotypes in Intermediate-Stage Hepatocellular Carcinoma after Transarterial Chemoembolization

**DOI:** 10.7150/jca.76021

**Published:** 2022-09-06

**Authors:** Siyu Chen, Aiping Guo, Linbin Lu, Shan Lin, Xinyu Hu, Lijun Zhu, Xi Chen

**Affiliations:** 1 Department of Oncology, the 900th Hospital of Joint Logistic Support Force, PLA, Fuzong Clinical College of Fujian Medical University, 350025, Fuzhou, Fujian, PR China.; 2 Department of Oncology, Mengchao Hepatobiliary Hospital of Fujian Medical University, 350025, Fuzhou, Fujian, PR China.; 3 Department of Neurology, the 900th Hospital of Joint Logistic Support Force, PLA, Fuzong Clinical College of Fujian Medical University, 350025, Fuzhou, Fujian, PR China.; 4 Fujian HCC-biomarker study group.

**Keywords:** Hepatocellular carcinoma, Transarterial chemoembolization, Subphenotype, Latent class analysis

## Abstract

**Background:** Transarterial chemoembolization (TACE) is the standard first-line therapy for intermediate-stage hepatocellular carcinoma (HCC). However, no latent-classing indices, concerning repeat conventional TACE or switching to another treatment, have been incorporated into the guidelines.

**Methods:** The unsupervised latent class modeling was applied to identify subphenotypes using the clinical and medical imaging data of 1517 HCC patients after the first TACE from four hospitals (derivation cohort: 597 cases; validation cohort: 920 cases); modeling was conducted independently in each cohort. We then explored the relationship of subphenotypes with clinical outcomes in both cohorts and response to treatment strategies after the first TACE in the derivation cohort.

**Results:** Independent latent class models suggested that a three-class model was optimal for both cohorts. In both cohorts, we identified a TACE-refractory subphenotype (Phenotype 1: PS score 1, stage progress, more intrahepatic lesions, and new intrahepatic lesions), TACE-responsive subphenotype (Phenotype 3: PS score 0, No intrahepatic lesions and new intrahepatic lesions), compared to TACE-intermediate subphenotype (Phenotype 2). Compared to Phenotype 1 or 2, patients in Phenotype 3 had significantly lower 3-month or 3-year mortality (all *P*<0.001). In the derivation cohort, the effects of treatment strategy (surgery/ablation vs. repeat TACE vs. stop TACE) differed significantly in phenotype 2 but not in phenotype 3 (*P*=0.721 for interaction).

**Conclusions:** Latent class models identified three subphenotypes for HCC after the first TACE treatment. Differences were significant in clinical outcome and response to treatment strategy after the first TACE among three subphenotypes.

## Introduction

Hepatocellular carcinoma (HCC), one of the most prevalent malignancies, is the major cause of cancer death worldwide, particularly in China 1. In other affected areas like Europe and the USA, transarterial chemoembolization (TACE) is taken as the mainstay of first-line treatment for intermediate-stage (BCLC B) HCC with unresectable tumors 2, which contains a population with a wide range of tumor burden and liver functions (Child-Pugh score 5-9). In real-world practice, TACE is not limited to treating BCLC B HCC. It is also applicable for early (BCLC A) HCC with surgery or radiofrequency ablation (FA) less effective and for advanced (BCLC C) HCC in combination with systematic therapy 3. However, this population has a variable median overall survival ranging from13 to 43 months 4. To further identify the population of BCLC B HCC patients who can benefit from TACE, plenty of tools for risk stratification are developed, including up-to-seven criteria 5, four-and-seven criteria 6, six-and-twelve criteria 7, HAP score 8, ALBI grade 9, and BCLC-B HCC sub-classification 10. After being highly selected through the method above, HCC patients have a median survival of 51.5 months 11, and their responses to TACE are still highly heterogeneous.

In clinical practice, the best sequential strategy is controversial for the intermediate stage HCC after TACE therapy. There are still no directions regarding the number of TACE performed before or when switching to another treatment strategy. For the purpose of guiding for a second TACE, some criteria, including the ART score 12, ABCR score 13, and SNACOR clinical scoring system 14, are developed to target the patients who benefit from further TACE sessions. Besides, when TACE is introduced as a preoperative therapeutic procedure, the response to TACE can be regarded as a criterion for the selection of liver resection 15. However, these classifications were based on the specific treatment. And it is far behind the requirements of emerging new therapies, such as sorafenib 16 or anti-PD-1 inhibitor 17 treatment after TACE failure. Whether subphenotypes existed within intermediate-stage HCC after TACE is urgent to explore. To deal with this issue, we perform latent class modeling to identify subphenotypes based on a large-scale multicenter HCC cohort.

## Methods and patients

### Patient Selection

Clinical and biochemical data were obtained from patients enrolled in the multicenter HCC cohort of Sun Yat-sen University 18-20. Details of this cohort study were previously described in full.

From January 2007 to December 2016, 2020, HCC patients with complete data were initially enrolled.

In this study, patients' clinical and biological data following TACE were collected at the second follow-up record, who had only one follow-up record were excluded (n=240). Patients who refused to receive treatment (n=37, 3.8%) and underwent surgery (n=225, 23.0%) as first-line therapy were excluded from the derivation cohort. In total, 120 patients with only one follow-up record were excluded, and finally 597 patients were included with TACE taken as the mainstay of treatment. Besides, analyses were repeated in an independent cohort (n=920, 65 patients excluded from the internal testing cohort and 55 patients from the multicenter testing cohort with only one follow-up record) to test whether the models could generalize to externally independent data. The following chart of patients selected as shown in Figure S1.

The study protocol (2017-FXY-129) and ethical issues 18, 21 of the present study had been published, which were waived for this secondary analysis study.

### Definition and Measurements

Overall survival (OS), the time from the beginning of the first TACE treatment to death by any cause, was regarded as the primary outcome indicator of this study. Stage progression-free survival referred to the time from diagnosis to the vascular invasion, distant or lymph node metastasis at the second follow-up visit. In the derivation cohort, the treatments after TACE included retreatment with TACE (re-TCAE, n=144, 24.1%), hepatic resection (HR, n=37, 6.2%), and radiofrequency or microwave ablation (RA, n=67, 11.2%). Furthermore, 349 patients (58.5%, including 3 with sorafenib), who received the best support therapy, were classified into the stop-TACE group.

This study's clinical and biochemical indices were at the second follow-up before any treatment. Vascular invasion only consisted of macroscopic vascular invasion, confirmed by the standard radiological imaging using at least two imaging modalities 22. Regarding the second follow-up record, new intrahepatic lesions were defined as new intrahepatic lesions rather than the residual lesions of the primary one within six months. Besides, the number of intrahepatic lesions were only the primary lesion's residual lesions after the first TACE treatment. Biochemical indices included serum alpha-fetoprotein (AFP) level, aspartate aminotransferase (AST) level, and Child-Pugh class (serum albumin, ALB; total bilirubin, TBIL).

### Statistical Analysis

The clinical data and biomarker levels at the second follow-up visit were taken as variables for class-defining in the LCA model. In contrast, clinical outcomes were not considered in the classification procedure. Other than the clinical data, five plasma biomarkers, AFP, AST, ALB, TBIL, and PT, were included. Statistical analyses for the LCA model were conducted using R depMixS4 23, a package inclusive of standard Markov models, latent/hidden Markov models, and latent class and finite mixture distribution models, with the expectation-maximization algorithm taken for parameter estimation.

First, we fitted a series of latent class models based on the derivation cohort and sequentially repeated them in the validation cohort independently. While for LCA model estimation, the full information maximum likelihood methods in the depMixS4 package were performed. This approach allowed all patients' data to estimate latent class models, including those with data missing. An optimal fit model selection was based on the following criteria: (1) the minimum of Bayesian Information Criteria (BIC) and the significant Vuong-Lo-Mendell-Rubin (VLMR) likelihood ratio test; (2) no less than 5% of participants in the smallest class size.

Next, after the 3-class model was identified, the differences in clinical, biochemical, and clinical outcomes were tested among three phenotypes with the number of classes determined. Besides, both cohorts drew the receiver operator characteristic (ROC) curve to select variables for phenotype prediction.

Finally, we evaluated models of each outcome for the derivation cohort with class, treatment assignment, and interaction as covariates to determine whether latent class-based differential therapeutic efficacy is present. Also, multivariable Cox proportional hazards models were adjusted for confounding factors.

Statistical significance was defined when a 2-tailed P-value was lower than 0.05. All analyses were completed with R 3.6.1 and Empower (www.empowerstats.com, X&Y solutions, Inc. Boston, MA).

## Results

### Baseline Characteristics of Cohorts at the Second Follow-Up Visit

From January 2007 to December 2016, 2020 patients suffering from BCLC B HCC from four study hospitals were initially enrolled. Based on the exclusion criteria, 1,517 patients with at least two follow-up records were involved (597 patients in the derivation cohort and 920 patients in the validation cohort). The majority of the patients were with HBV infection 18. The derivation cohort had more intrahepatic lesions, larger tumor diameter, and a higher proportion of two lobes with lesions than the validation cohort (Table l). Baseline demographic, hematological, and medical imaging data of the 1,517 subjects were detailed in Table S1.

Overall, 58.8% (n=351) of patients in the derivation cohort died, and 42.8% (n=394) of patients in the validation cohort at the deadline of this study. The median OS was 16.2 months (0.9-115.3) for the derivation cohort and 19.2 months (0.9-98.5) for the validation cohort. Besides, the median cumulated time since the first admission was 2.1(0.4 - 8.0) /2.0(0.1 - 8.0) months for the derivation/validation cohort, respectively.

### Identification of Number of Phenotypes for Latent-Class Modeling

The latent-class models of each cohort indicated that the optimal fit was achieved with a three-class model (see Table S2 for summarized model fits of both cohorts for 2 through 5 classes). In the three-class model, the mean latent class probabilities for the most probable class for Phenotypes 1, 2, and 3 in the derivation cohort were all 1.0000. Similarly, the probabilities in the validation cohort were all 1.0000 for three sub-phenotypes. It demonstrated a class assignment with a good model fit and highly strong probabilities.

### Clinical and Biological Characteristics of Each Phenotype

**Figure 1:**
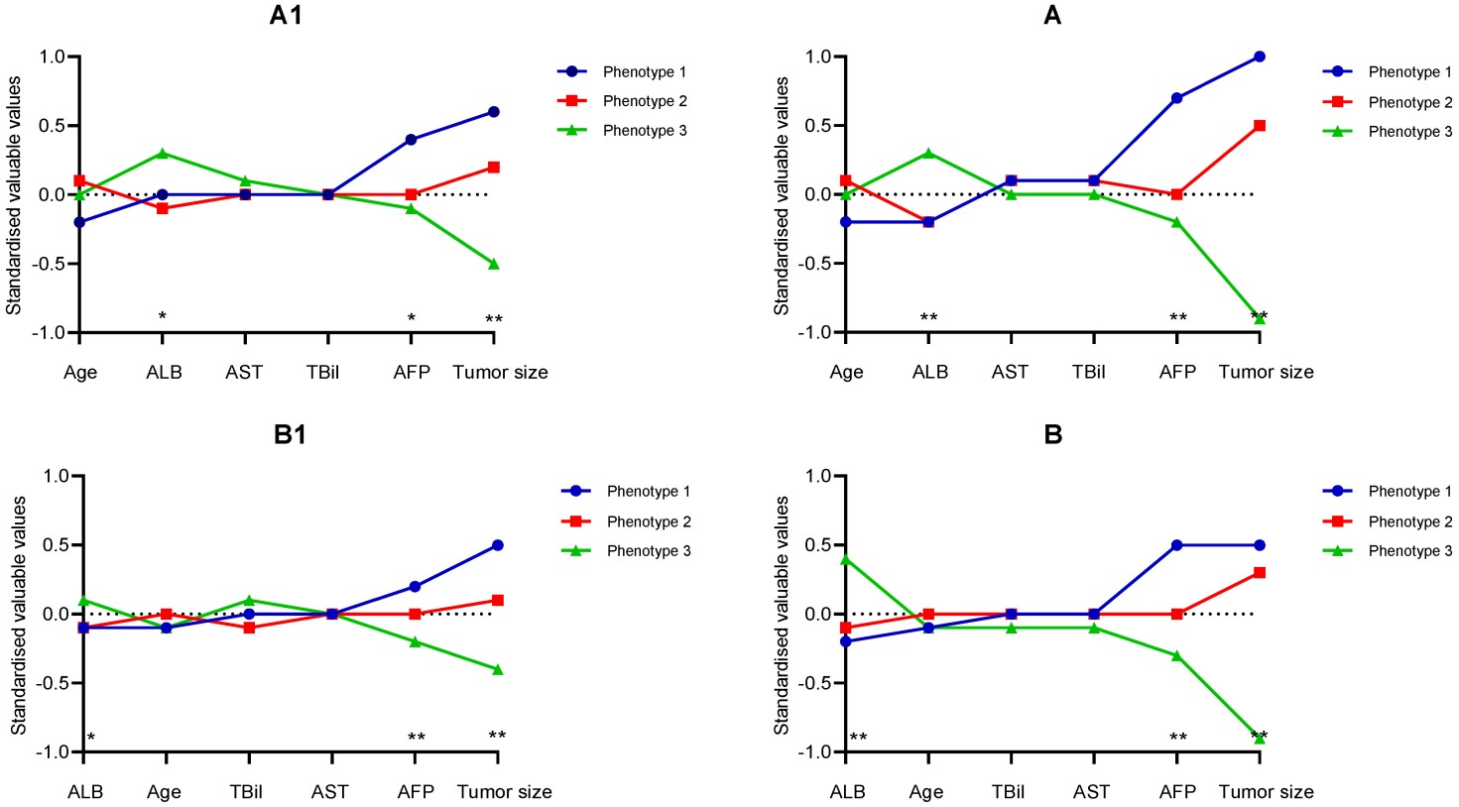
Differences in each variable's standardized values by phenotype on the y-axis, with the individual continuous variables along the x-axis, for the derivation cohort (Figure 1A) and the validation cohort (Figure 1B). Figure 1 A1/B1 refer to variables before first TACE, and Figure 1 A2/B2 after TACE. The variables are sorted based on the degree of separation between the classes from the maximum positive separation on the left to the maximum negative separation on the right. Variable standardization is scaled to zero and standard deviations to one; a value of +1 for the standardized variable signifies that the mean value for a given phenotype was one standard deviation higher than the mean value in the cohort whole.

The clinical and biological features which could be taken to classify each phenotype were sequentially discussed. Following an assignment over the most likely phenotype of participants, variables used for each phenotype were examined and averaged. The continuous variables examined in the derivation cohort were managed by the separation degree between the phenotypes. As displayed in Figure 1 A1, Phenotype 1 had significantly higher AFP, ALB level, and larger main tumor relative to Phenotype 1/2 in the derivation/validation cohort. After TACE treatment, a similar relationship was found in both cohorts (Figure 1 A2).

Furthermore, we could find that major tumor size and log AFP had the largest degree of separation. Additionally, the differences concerning the categorical variables were presented in Table 2. It could be seen that there were no intrahepatic lesions or new intrahepatic lesions, well performance status (PS 0) in Phenotype 3. Moreover, the patients of Phenotype 1 had stage progress and poor performance status (PS 1), with the highest percentage of diameter of main tumor over 5 cm, *No.* intrahepatic lesions more than three and new intrahepatic lesions, while the patients of Phenotype 2 had the average percentage of diameter of main tumor over 5 cm, *No.* intrahepatic lesions more than 3, PS 1, and new intrahepatic lesions.

As described in the methods, the latent-classing models in the validation cohort were repeated independently. The contribution of key variables was presented in Figure 1 A2/B2. It revealed that there were significant similarities in the features of the three subphenotypes between the validation cohort and derivation cohort, with one phenotype (Phenotype 3) characterized by no intrahepatic lesions and new intrahepatic lesions, well performance status (PS 0), and compared to the other two phenotypes (Phenotype 2 and 3), which was shown in Table 2. Specifically, the primary lesions in Phenotype 1 had the optimal response to TACE treatment with minimum tumor burden in both cohorts.

### Phenotype Prediction with Reduced Number of Variables

To determine whether phenotype prediction can be effectively realized by using a reduced number of variables, measures with the highest difference in mean absolute values between phenotypes in the derivation cohort were taken as predictors for ROC analysis. Three variables, including PS score (0/1), *No.* intrahepatic lesions (0/≤3/>3), new intrahepatic lesion (no/yes), stage progress (no/yes), were considered. From the results of the ROC analysis, the area under the curve (AUC) for phenotype prediction was 1.000 in the whole cohort, suggesting that phenotype could be accurately predicted with a modest number of variables. The decision tree of phenotype was shown in Figure 3.

### Association between Phenotype and Clinical Outcomes

**Figure 2:**
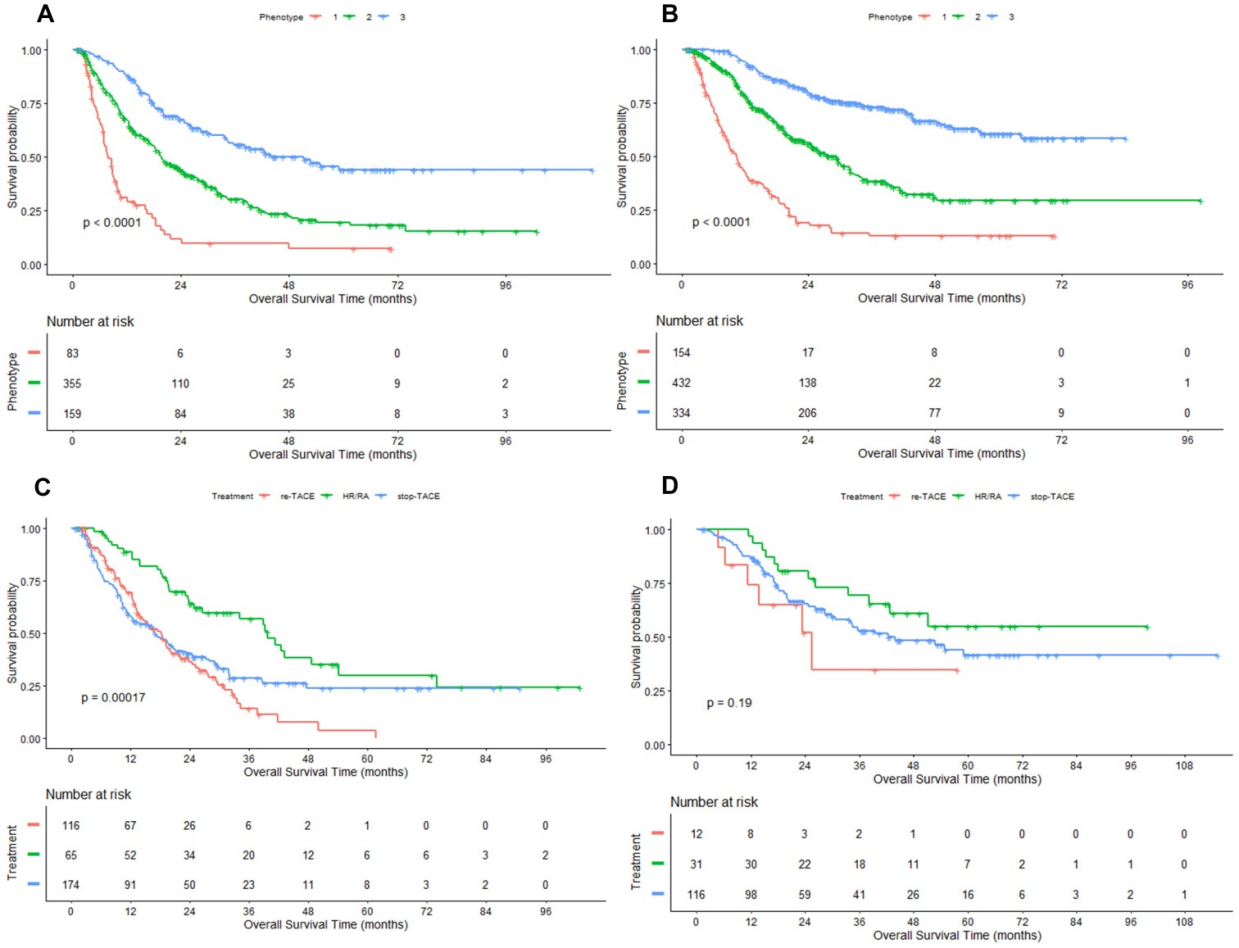
** Kaplan-Meier curves of OS in HCC patients treated with first-line TACE.** Figure 2 A/B: derivation/ validation cohorts; Figure 2 C/D: Phenotype 2/3 in the derivation cohort. HR: hepatic resection; RA: radiofrequency/microwave ablation.

**Figure 3:**
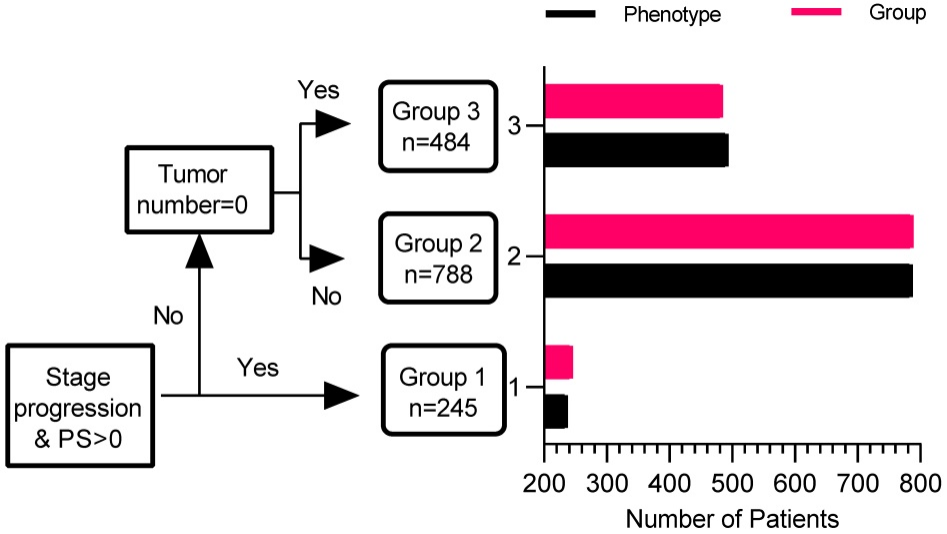
** Decision tree of phenotype with four key valuables.** The red bar is determined by the decision tree, and the black bar is determined by latent class analysis.

To determine whether there are varying natural histories among the three phenotypes, we conducted an association analysis for probable phenotype assignment and clinical outcomes. For the Phenotype 1 to 3, the median OS was 7.8 (95% CI: 6.9, 9.8), 19.6 (17.5, 23.9) and 51.2 (34.4, NA) months in the derivation cohort (Figure 2A), and 10.4 (95%CI: 8.7, 12.3), 29.3 (24.5, 32.0) months and not reached in the validation cohort (Figure 2B), respectively. In the derivation cohort, subjects in Phenotype 1 had significantly higher 3-month and 3-year mortality compared with subjects in Phenotype 2 and 3 (6.0% vs. 2.3% vs. 0.6% for 3-month mortality; 72.3% vs. 57.5% vs. 39.0% for 3-year mortality; All *P* < 0.01). Likewise, similar results were observed in the validation cohort (6.5% vs. 1.2% vs. 0.0% for 3-month mortality; 68.8% vs. 43.1% vs. 23.1% for 3-year mortality; all *P* < 0.001).

Compared with Phenotype 1, hazard ratio for Phenotype 2 was 0.40 (95% CI: 0.33, 0.48) and 0.16 (95% CI: 0.13, 0.20) for Phenotype 3 in the whole cohort. Because the valuables determined the 3-class model at the second follow-up record, hazard ratios (Phenotype 3: 0.24, 95%CI: 0.18, 0.30; Phenotype 2: 0.48, 95% CI: 0.39, 0.58) were further adjusted by baseline characteristics before first TACE, including age, gender, Child-Pugh class (A, B), LogAFP, No. of intrahepatic lesions (2, 3, >3), Diameter of main tumor, Location of lesions (left, right, both).

### Treatment Strategy on Clinical Outcomes Stratified by Phenotype at the Second Follow-up Visit

At last, we determined the differences in response to the following treatments based on phenotype using the data from the derivation cohort. In the overall cohort, patients who underwent HR or RA enjoyed a highly better clinical outcome compared to the patients who stopped TACE and received TACE treatment, with the median OS of 42.5 (95%CI: 34.1, NA), 19.5 (95%CI: 17, 24.5), and 16.2 (95%CI: 13.3, 19.8) months, respectively. We found that the treatment strategy (re-TACE, HR/FA, stop TACE) had no significantly different survival effects between the phenotype 2 and 3 (*P*=0.721 for interaction). Only in Phenotype 2 the differences between the three phenotypes were significant (P=0.002, Figure 2C).

## Discussion

The latent-class models identified the three subphenotypes before the planned dual therapy after the first TACE in this large-scale, multicenter cohort study. This three-class model could be accurately predicted with four key variables: PS score, *No.* intrahepatic lesions, new intrahepatic lesions, and stage progress. Furthermore, subphenotypes were strongly associated with clinical outcomes, with significant differences in mortality at three months and three years. In both cohorts, although the differences were substantial in baseline characteristics, we identified a TACE-refractory subphenotype (Phenotype 1: PS score 1, stage progress, more intrahepatic lesions, and new intrahepatic lesions), TACE-responsive subphenotype (Phenotype 3: PS score 0, No intrahepatic lesions and new intrahepatic lesions), compared to TACE-intermediate subphenotype (Phenotype 2).

Recently, some scoring systems 12-14 have been developed to support decision-making after the first TACE, dividing the patients into two groups (well vs. poor prognosis). Nevertheless, only a small amount of patients were suitable to repeat TACE treatment. In our study, stop TACE was superior to repeat TACE in the whole cohort. A possible reason was that not all patients treated with repeat TACE were the optimal population. On the other hand, switching to another treatment (e.g., target therapy 24) would provide a more favorable outcome for those who do not benefit from TACE. In the derivation cohort, the interaction between subphenotype 2/3 and treatment strategy (re-TACE, HR/FA, stop TACE) was not significant. Although current evidence was not enough to prove the best treatment (HR/RA vs. repeat vs. stop TACE) in the TACE-responsive subphenotype, HR/RA was optimal for patients in the TACE-intermediate subphenotype. Besides, predictive variables differed in the ART score 12 (increase in Child-Pugh score from baseline, AST increase >25%, radiologic tumor response), ABCR score 13 (BCLC and AFP at baseline, increased Child-Pugh score by ≥ 2 from baseline, and the radiological response) and SNACOR score 14 (tumor size, tumor number, baseline AFP level, Child-Pugh class, objective radiological response). However, this study identified a three-class model using unsupervised latent class analysis, with four key variables (PS score, intrahepatic lesions number, new lesions, and stage progress). These factors could accurately predict the LCA model's subphenotypes, with an AUC value of 1.000 in both cohorts.

The present study had several advantages. For instance, this study involved a large-scale cohort from four hospitals in south China. Under patients enrolled in the multicenter cohort, the samples studied reflect demographically diverse immediate stage HCC cohorts. Though the baseline data were significantly different between the two groups, identifying three phenotypes was robust and independent in the derivation and validation cohort. It strengthened the generalizability of our findings and the similarity of the subphenotypes identified in the two cohorts. Second, this was the first unsupervised classification after TACE based on the latent-classing model. Likewise, since clinical outcomes were out of the variables for class-defining, the strengths and consistency regarding the relationship between subphenotypes and clinical outcomes are striking. Third, treatment strategy data were collected before the repeated TACE scoring systems 12-14 were developed, minimizing selective bias. This three-class model would be a crucial supplement to current scoring systems.

There still exist some limitations in this study. For example, the patients enrolled in our study were from real-world practice in the south of China, which may lead to the diversity of the subphenotypes in the randomized controlled trials or the western populations. Besides, the biochemical indices were limited to those already examined in both cohorts. Even though those four key biomarkers were valuable in accurate phenotype prediction, other informative data were unknown in this study, including the cirrhosis rate, portal hypertension, and MELD score. Besides, we would commit to developing and validating a predictive model to determine which phenotype the patients belong to in the future.

In summary, our analysis identified a three-class model within two independent cohorts of HCC patients following TACE treatment. The three subphenotypes of the model are markedly diverse in clinical and biological features, clinical outcomes, and treatment responses.

## Supplementary Material

Supplementary figure and tables.

## Figures and Tables

**Table 1:** Comparison of key clinical data points between derivation and validation cohorts after first TACE treatment

	Derivation cohort (n=597)	Validation cohort (n=920)	*P*-value
**Age (yr)**			0.015
<55	283 (47.4%)	495 (53.8%)	
≥55	314 (52.6%)	425 (46.2%)	
**Gender**			<0.001
male	547 (91.6%)	427 (46.4%)	
female	50 (8.4%)	493 (53.6%)	
**PS score**			0.588
0	490 (82.1%)	765 (83.2%)	
1	107 (17.9%)	155 (16.8%)	
**AST (U/L) , missing data=135**			0.005
<45	254 (47.4%)	466 (55.1%)	
≥45	282 (52.6%)	380 (44.9%)	
**Child-Pugh class, missing data=810**			0.362
A	53 (17.1%)	62 (15.6%)	
B	253 (81.6%)	324 (81.6%)	
C	4 (1.3%)	11 (2.8%)	
LogAFP (ng/mL), missing data=120	2.2 ± 1.4	1.9 ± 1.4	0.001
***No.* of intrahepatic lesions**			<0.001
0	159 (26.6%)	354 (38.5%)	
<3	155 (26.0%)	217 (23.6%)	
≥3	283 (47.4%)	349 (37.9%)	
**Diameter of main tumor (cm)**			<0.001
0	159 (26.6%)	358 (38.9%)	
<5	155 (26.0%)	234 (25.4%)	
≥5	283 (47.4%)	328 (35.7%)	
**Location of lesions**			<0.001
none	159 (26.6%)	355 (38.6%)	
left/right	163 (27.3%)	237 (25.8%)	
both	275 (46.1%)	328 (35.7%)	
**New intrahepatic lesions**			0.964
no	506 (84.8%)	779 (84.7%)	
yes	91 (15.2%)	141 (15.3%)	
**Vascular invasion**			0.916
no	552 (92.5%)	852 (92.6%)	
yes	45 (7.5%)	68 (7.4%)	
**Distant metastasis**			0.903
no	544 (91.1%)	840 (91.3%)	
yes	53 (8.9%)	80 (8.7%)	
**Lymph node metastasis**			0.897
no	561 (94.0%)	866 (94.1%)	
yes	36 (6.0%)	54 (5.9%)	

Numbers that do not add up to 597 or 920 are attributable to missing data.

**Table 2:** Differences in variables based on phenotype assignment in the derivation and validation cohorts after the first TACE treatment

Phenotype	Derivation cohort			Validation cohort		
	1	2	3	1	2	3
N	83	355	159	154	432	334
**Gender**						
male	76 (91.6%)	320 (90.1%)	151 (95.0%)	69 (44.8%)	176 (40.7%)	182 (54.5%)
female	7 (8.4%)	35 (9.9%)	8 (5.0%)	85 (55.2%)	256 (59.3%)	152 (45.5%)
Child-Pugh class*, missing data =95						
A	66(80.5%)	298 (87.9%)	144 (92.3%)	118 (78.7%)	343 (88.4%)	270 (87.9%)
B	16(19.5%)	41 (12.1%)	12 (7.7%)	32 (21.3%)	45 (11.6%)	37 (12.1%)
AFP (ng/ml)*, missing data =85						
<200	24 (29.6%)	164 (48.8%)	77 (50.3%)	48 (32.4%)	196 (49.0%)	161 (51.3%)
≥200	57 (70.4%)	172 (51.2%)	76 (49.7%)	100 (67.6%)	204 (51.0%)	153 (48.7%)
**PS score**						
0	0(0.0%)	344 (96.9%)	146 (91.8%)	0 (0.0%)	431 (99.8%)	334 (100.0%)
1	83(100.0%)	11 (3.1%)	13 (8.2%)	154 (100.0%)	1 (0.2%)	0 (0.0%)
**Diameter of main tumor (cm)**						
0	0 (0.0%)	0 (0.0%)	159 (100.0%)	24 (15.6%)	0 (0.0%)	334 (100.0%)
<5	17 (20.5%)	138 (38.9%)	0 (0.0%)	37 (24.0%)	197 (45.6%)	0 (0.0%)
≥5	66 (79.5%)	217 (61.1%)	0 (0.0%)	93 (60.4%)	235 (54.4%)	0 (0.0%)
**No. of intrahepatic lesions**						
0	0 (0.0%)	0 (0.0%)	159 (100.0%)	24 (15.6%)	0 (0.0%)	334 (100.0%)
≤3	14 (16.9%)	141 (39.7%)	0 (0.0%)	29 (18.8%)	188 (43.5%)	0 (0.0%)
>3	69 (83.1%)	214 (60.3%)	0 (0.0%)	101 (65.6%)	244 (56.5%)	0 (0.0%)
**New lesions**						
no	44 (53.0%)	309 (87.0%)	159 (100.0%)	93 (60.4%)	353 (81.7%)	334 (100.0%)
yes	39 (47.0%)	46 (13.0%)	0 (0.0%)	61 (39.6%)	79 (18.3%)	0 (0.0%)
**Stage progression**						
no	0 (0.0%)	354 (99.7%)	150 (94.3%)	0 (0.0%)	431 (99.8%)	332 (99.4%)
yes	83 (100.0%)	1 (0.3%)	9 (5.7%)	154 (100.0%)	1 (0.2%)	2 (0.6%)

*Before first TACE. Numbers that do not add up to 597 or 920 are attributable to missing data.

## Abbreviations

TACE: transarterial chemoembolization (TACE)
HCC: hepatocellular carcinoma
BCLC: Barcelona Clinic Liver Cancer
HAP: Hepatoma Arterial-embolization Prognostic
ALBI: albumin-bilirubin
ART: Assessment for Retreatment with TACE
ABCR: alpha-fetoprotein, BCLC, Child-Pugh, and response
SNACOR: tumor Size, tumor Number, baseline Alpha-foetoprotein level, Child-Pugh class, and Objective radiological Response
LCA: latent class analysis
AST: aspartate aminotransferase
AFP: alpha-fetoprotein
BIC: Bayesian Information Criteria
ROC: receiver operator characteristic
