# Supplementary material for: Latent Class Analysis of Subphenotypes in Intermediate-Stage Hepatocellular Carcinoma after Transarterial Chemoembolization
Source: J Cancer. 2022 Sep 6;13(12):3318–25. doi: 10.7150/jca.76021 (PMC9516009; doi:10.7150/jca.76021)
Supplement: Supplementary file 1 — Supplementary figure and tables. [file jcav13p3318s1.pdf]

Supplementary materials

Table S1. Baseline characteristics of patients among three cohorts before first TACE treatment.

	derivation cohort	internal testing cohort	multicenter testing cohort	P-value
N	597	562	358	
Age	53.3 ± 12.2	53.3 ± 11.7	51.2 ± 12.0	0.014
Gender				<0.001
male	547 (91.6%)	130 (23.1%)	297 (83.0%)	
female	50 (8.4%)	432 (76.9%)	61 (17.0%)	
ALB (g/L), missing data=23	38.9 ± 5.6	38.9 ± 4.9	38.8 ± 5.7	0.946
Log TBil (umol/L) , missing data=46	1.3 ± 0.2	1.3 ± 0.3	1.3 ± 0.3	0.040
Log AST (U/L), missing data=23	1.9 ± 0.4	1.9 ± 0.4	1.8 ± 0.4	0.206
Child-Pugh class, missing data =95				0.499
A	508 (88.0%)	452 (87.3%)	279 (85.3%)	
B	69 (12.0%)	66 (12.7%)	48 (14.7%)	
AFP (ng/ml), missing data=85				0.575
<200	265 (46.5%)	235 (45.5%)	170 (49.1%)	
≥200	305 (53.5%)	281 (54.5%)	176 (50.9%)	
Diameter of main tumor(cm）	7.3 ± 3.7	7.0 ± 3.4	7.1 ± 3.5	0.286
Location of Lesions				<0.001
left lobe	14 (2.3%)	39 (6.9%)	19 (6.1%)	
right lobe	204 (34.2%)	212 (37.7%)	121 (38.9%)	
both lobe	379 (63.5%)	311 (55.3%)	171 (55.0%)	
No. of intrahepatic lesions				0.004
2	148 (24.8%)	182 (32.4%)	128 (35.8%)	
3	47 (7.9%)	44 (7.8%)	21 (5.9%)	
>3	402 (67.3%)	336 (59.8%)	209 (58.4%)	

Numbers that do not add up to 597 or 562 or 358 are attributable to missing data.

Table S2. Fit statistics for latent class models from two to five classes.

Number of classes	BIC	N1	N2	N3	N4	N5	P-value
**Derivation cohort**							
2	3658.95	506 (84.8%)	91 (15.2%)				<0.000001
3	3034.69	83 (13.9%)	355 (59.5%)	159 (26.6%)			<0.000001
4	3051.18	83 (13.9%)	14 (2.3%)	355 (59.5%)	145 (24.3%)		<0.000001
5	3426.82	104 (17.4%)	9 (1.5%)	150 (25.1%)	251 (42%)	83 (13.9%)	<0.000001
**Validation cohort**							
2	9353.91	562 (61.1%)	358 (38.9%)				<0.000001
3	9296.62	130 (14.1%)	432 (47%)	358 (38.9%)			<0.000001
4	8777.69	130 (14.1%)	26 (2.8%)	432 (47%)	332 (36.1%)		<0.000001
5	8881.47	432 (47%)	26 (2.8%)	332 (36.1%)	93 (10.1%)	37 (4%)	<0.000001

#By Vuong-Lo-Mendell-Rubin likelihood ratio test, testing whether the number of classes provides an improved model fit compared to a model using one fewer class.

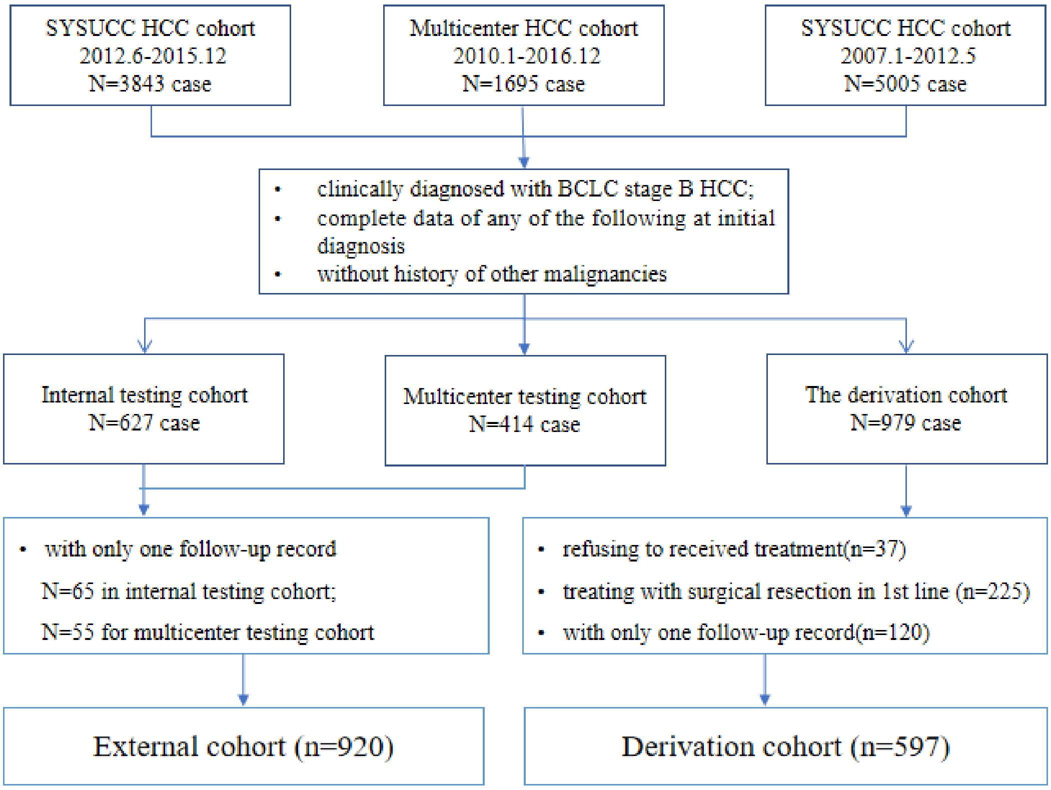

Figure S1. Flowchart for the patients with HCC after first TACE treatment. Between January 2007 and May 2012, 5005 consecutive patients with newly diagnosed HCC at Sun Yat-sen University Cancer Center (SYSUCC) were retrospectively reviewed to develop the derivation cohort. Between June 2012 and December 2015, a consecutive independent series of 3843 HCC patients treated at SYSUCC were examined to establish the internal testing cohort. Besides, between January 2010 and December 2016, 843 patients from Fifth Affiliated Hospital of Sun Yat-sen University, 415 patients from the Third Affiliated Hospital of Sun Yat-sen University, and 437 patients from the Second Hospital of Guangzhou Medical University were reviewed to develop the multicenter testing cohort. After meeting the inclusion criteria, a total of 979, 627, and 414 patients were included in the derivation cohort, internal testing cohort, and multicenter testing cohort, respectively. According to the exclusion criteria, 597 and 920 patients were included in the derivation cohort and validation cohort, respectively.
